# Obtaining miRNA from Saliva—Comparison of Sampling and Purification Methods

**DOI:** 10.3390/ijms24032386

**Published:** 2023-01-25

**Authors:** Aintzane Urbizu, Laura Arnaldo, Katrin Beyer

**Affiliations:** 1 Department of Pathology, Germans Trias i Pujol Research Institute (IGTP), 08916 Badalona, Spain; 2 Department of Biochemistry, Universitat Autònoma de Barcelona, 08193 Barcelona, Spain

**Keywords:** miRNAs, saliva biomarker, purification methods, miRNA quantification

## Abstract

The use of saliva as a biomarker source has advantages over other biofluids and imaging techniques, and miRNAs are ideal biomarker candidates. They are involved in numerous cellular processes, and their altered expression suggests that miRNAs play a crucial regulatory role in disease development. We wanted to find an easily reproducible and executable miRNA-obtaining methodology suitable for quantification. Three commercial miRNA extraction kits (mirVana, Nucleospin and miRNeasy) and three saliva collectors (50 mL tubes, Salimetrics and Oragene) were tested. Several features, including RNA quality and technical parameters, were evaluated. The expression of five synthetic spike-in controls and seven saliva-miRNAs was analyzed independently and grouped by the collectors and the extraction kits. The combination of Oragene and miRNeasy assured the most sensitive detection of all seven saliva miRNAs. Testing different combinations of saliva collectors and RNA purification kits permitted the establishment of combinations for different uses. The results of our study highlight that optimization of resources for biomarker studies is possible after careful planning of each study.

## 1. Introduction

Non-coding RNAs (ncRNA) are RNA molecules that are not translated into a protein. ncRNAs are divided into small ncRNAs (<200 nt) that include miRNAs, piRNAs, snoRNAs; and long ncRNAs (lncRNA) (>200 nt). miRNAs, in particular, have a length average of 22 nt and play important roles in regulating gene expression by affecting the translation and stability of their mRNA targets through binding to the 3′ untranslated region (UTR) in most cases, although interacting with 5′UTRs, coding sequences and gene promoters have also been reported [1]. Mature miRNAs can interact with their target RNAs in subcellular locations, or be secreted into extracellular fluids, such as plasma and serum, cerebrospinal fluid, saliva, breast milk, urine, tears or peritoneal fluid. They can also be transported to target cells via extracellular vesicles (exosomes) or by binding to proteins such as argonaute-2, nucleophosmin-1 and high-density lipoprotein [1,2]. 

miRNAs are involved in numerous cellular processes in different tissues and organs, including cell proliferation, differentiation, apoptosis, energy balance, metabolic homeostasis, inflammation, angiogenesis and DNA repair. Altered expression of certain miRNAs suggests that they could have a crucial regulatory function in disease development [3]. Therefore, extracellular miRNAs might represent useful biomarkers for a variety of disorders such as cancer, epilepsy or neurodegenerative, autoimmune and mitochondrial diseases [2]. In fact, in the last decade numerous studies have focused on the identification of miRNAs as blood, serum, CSF or saliva biomarkers to facilitate disease diagnosis and monitoring, including treatment response. However, the lack of reproducibility of the findings indicates that the use of miRNAs as biomarkers is still in its early stages [2].

Factors such as ageing can directly affect and modulate miRNA expression levels. The expression of miRNAs found in biological fluids, called circulatory miRNAs, might correlate with aging and lifespan in humans. Since circulatory miRNAs can be easily obtained, they could be used as noninvasive biomarkers of aging and for tracking individual decline [4]. In plasma, three ageing peaks producing significant changes in the metabolome and transcriptome have been described to occur around the ages of 34, 60 and 75 [5]. Correspondingly, a signature of decreased miRNAs was identified in individuals older than 60 years compared to individuals 30 years of age, leading to an increased expression of ageing-related gene sets [6].

Another modifying factor of miRNA expression in biofluids is the circadian rhythm. In this context, defined miRNA sets showing consistent diurnal oscillation have been identified in both human plasma and saliva and have been denominated CircaMiRs [7,8]. Because miRNAs regulate the majority of human genes, a considerable number of circadian genes are now thought to be directly regulated by miRNAs [9].

Finally, lifestyle and environmental factors have also been shown to exert a direct influence on miRNA expression. Among several factors, long-term exercise significantly alters the profiles of plasma miRNAs [10], and both aerobic and resistance-based exercise induces miRNA expression changes [11]. When analyzing the role of tobacco in fetal growth, specific miRNAs were identified to reduce growth when smoking during pregnancy [12].

Specifically, in saliva, the transcriptome of the microbiome represents an elevated percentage of total RNA with pronounced inter-individual heterogeneity [13]. The higher the RNA content corresponding to the microbiome, the higher the masking rate of expression data [13,14].

The use of saliva as a biomarker source has advantages over other biofluids and imaging techniques because its collection is noninvasive, inexpensive and requires minimal personnel training [15,16,17]. Saliva is an ultrafiltrate of blood plasma and therefore mirrors systemic processes, mainly via extracellular vesicles, including exosomes [13,14]. The latter are excreted from virtually all organs and carry information on ongoing pathological changes in the organism [18,19].

However, few studies have explored the utility of saliva miRNAs as disease biomarkers. One of the reasons could be the lack of specific commercial kits to extract miRNA from saliva, so the protocols of other extraction kits have to be adjusted [20,21]. Furthermore, since there are no standardized protocols for sample collection, storage or methodology, every researcher uses the most convenient method. Therefore, the implementation of each step described in the literature has several possible outcomes, without evidence of which is the best option.

To overcome this issue, we wanted to determine which methodology for whole saliva miRNA obtaining samples suitable for quantification is easily reproducible and executable. Therefore, we tested and compared three different commercial miRNA extraction kits in combination with three different saliva collectors to determine the most suited protocol regarding efficiency, time and cost to be considered for future studies.

## 2. Results

**Figure 1:**
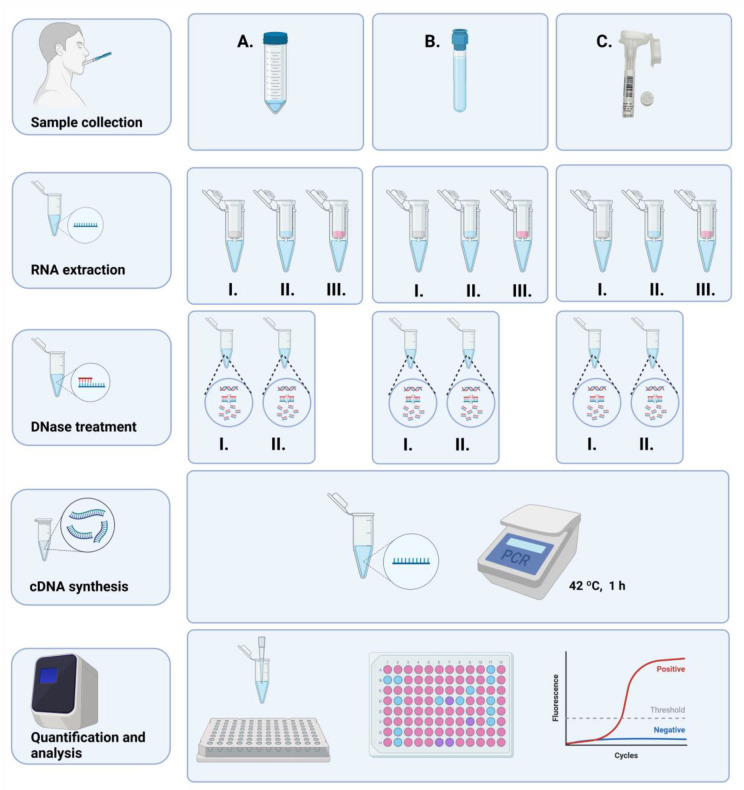
Scheme of the study. In sequential order, from top to bottom, saliva collection (three different collectors, (**A**) 50 mL tubes, (**B**) Salimetrics, (**C**) Oragene), RNA extraction (three different kits, I. mirVana, II. Nucleospin, III. miRNeasy), DNase treatment (RNA samples purified with I. mirVana, II. Nucleospin), cDNA synthesis (for 1 h at 42 °C), miRNA quantification on a real-time PCR machine. Created in BioRender.com, accessed on 29 November 2022.

The schematic representation of the study, starting with the collection of samples until miRNA quantification, is illustrated in Figure 1.

### 2.1. Saliva Collectors

Three different saliva collectors were tested, Oragene RNA collection tubes (Oragene), Salimetrics 2 mL cryovials (Salimetrics) and 50 mL sterile conical tubes (50 mL tubes). Regarding the saliva collection procedure, three of the five volunteers reported major difficulty in collecting saliva into the Salimetrics collector, although it included the Saliva Collector aid specifically designed for that use. Oragene saliva collectors were the most expensive. The use of 50 mL tubes represents only 2.5% of the cost of Salimetrics collectors, or 12.5% of the cost of Oragene collectors. When taking into account the three saliva collectors, although 50 mL tubes and Salimetrics tubes are processed or stored immediately after saliva obtaining, the Oragene collector has to be incubated before processing at 50 °C for one hour in a water bath, or two hours in an air incubator.

### 2.2. miRNA Extraction Kits

Three different kits were used, the mirVana miRNA isolation kit (MV kit), the NucleoSpin^®®^ miRNA Plasma kit (NS kit) and the miRNeasy Serum/Plasma Advanced Kit (MR kit). Regarding the time cost of the miRNA extraction protocol, the three RNA purification kits used a similar amount of time, approximately 1.5 h, for the processing of two or three samples. Regarding the economic cost, MV was the most expensive kit, allowing users to process only 20 samples. However, MV is the only kit that allowed the obtaining of total RNA and small RNAs in separated fractions. In comparison, the cost of MR, including the DNase kit, would be approximately 60% of the cost of MV, and NS, also including the DNase kit, would be 40% of the cost of MV.

As a result, the most economical combination would consist of using 50 mL tubes with the NS kit, and the most expensive would be using Oragene collectors and the MV kit.

### 2.3. RNA Extraction

#### 2.3.1. Total RNA Amount

**Figure 2:**
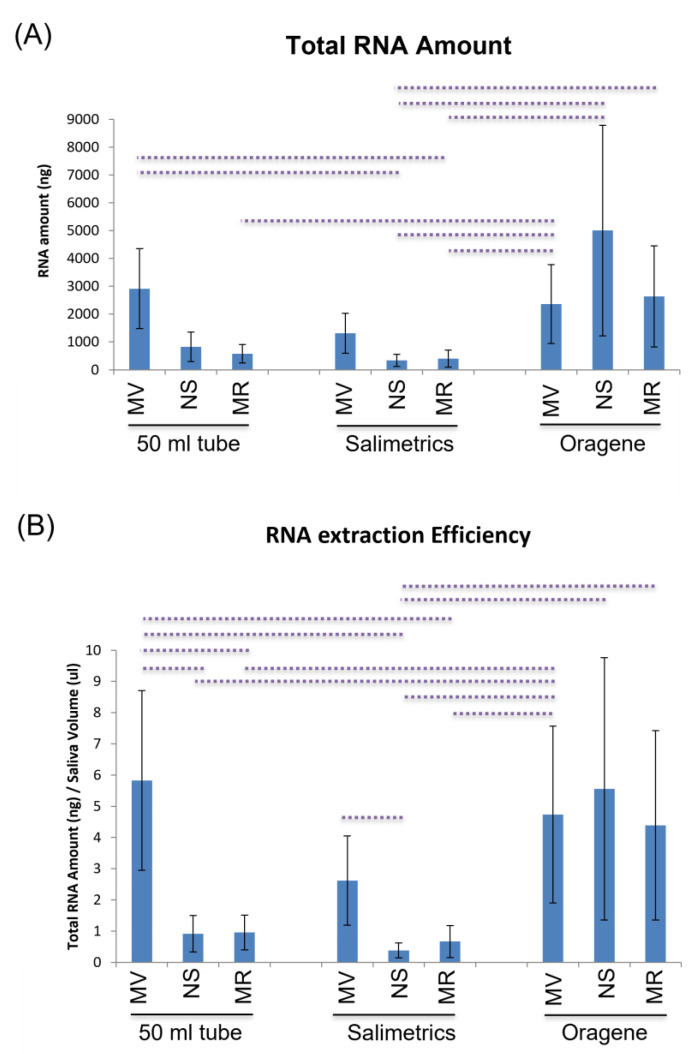
RNA purification amount and efficiency. Vertical bar chart shows the comparison between the combination of the three saliva collectors (50 mL tube, Salimetrics and Oragene) with three miRNA extraction kits, MV (mirVana miRNA isolation kit), NS (Nucleospin miRNA Plasma kit), MR (miRNeasy Serum/Plasma Advanced kit). (**A**) Amount of total RNA (in ng) isolated from whole saliva. (**B**) RNA extraction efficiency (ng of total RNA per µL of saliva used). Dashed lines represent significant differences (*p* < 0.025).

Total RNA was extracted using three different kits in combination with three different saliva collectors from 15 samples. Whereas for the NS and MR kits only total RNA samples were obtained, the MV kit could purify a separate fraction of small RNAs leading to 60 miRNA/total RNA samples (Table S1). When comparing the saliva collectors, the RNA amount obtained using Oragene collectors, independently of the miRNA extraction kit was significantly higher than with the other collectors (10.01 μg for Oragene vs. 4.31 μg for 50 mL tubes and 2.05 μg for Salimetrics, *p* < 0.002). The RNA amounts obtained using NS and MR in combination with Oragene were strikingly higher than those obtained with the same kits but in combination with either 50mL tubes or Salimetrics (Figure 2A, Table S1). In fact, the combination of Oragene and NS allowed us to obtain the highest amount of total RNA, 5003 ng. When comparing miRNA extraction kits, no significant differences were observed in the total RNA amounts (*p* > 0.05). However, the use of the MV kit resulted in obtaining an elevated amount of total RNA in combination with all three collectors: 2914 ng with 50 mL tubes, 1311 ng with Salimetrics and 2365 ng with Oragene. These amounts were significantly higher when combined with Oragene or the 50 mL tube in comparison with the NS and MR combined with the 50 mL tube or Salimetrics (Figure 2A, Supplementary Materials Table S2). The RNA amounts obtained with the NS kit were 825 ng with 50 mL tubes, 340 ng with Salimetrics and 5003 ng with Oragene; and with the MR kit, 575 ng with 50 mL tubes, 402 ng with Salimetrics, and 2636 ng with Oragene (Figure 2A).

#### 2.3.2. Efficiency

Oragene seemed to be the most efficient collector of RNA (4.89 ng/μL, Oragene vs. 2.57 ng/μL, 50 mL tube and 1.22 ng/μL, Salimetrics; *p* < 0.002) and MV the most efficient kit (2.82 ng/μL, MV vs. 2.28 ng/μL, NS and 2.00 ng/μL, MR; *p* < 0.05). In fact, as with the total RNA amount, the use of the MV was significantly more efficient when combined with Oragene or the 50 mL tube in comparison with the NS and MR combined with 50mL tubes or Salimetrics (Figure 2B, Table S1). The combinations of 50mL tubes and MV, and Oragene and NS gave the most total RNA (including miRNAs) per µL of saliva: 5.83 and 5.56 ng/µL, respectively. Similar efficiencies were obtained using Oragene in combination with either MV or MR: 4.73 and 4.39 ng/µL, respectively (Figure 2B, Table S3).

#### 2.3.3. Purity

The purity of the RNA samples was addressed after measuring the absorbance at 260 nm and 280 nm. RNA samples obtained with the MR kit had the highest purity as determined by the A260/A280 ratio, independent of the collector tube. When using 50mL tubes, 80% of the RNA samples had ratios between 1.8 and 2.1, when using Salimetrics, 50% and 60% when using Oragene (Table S1).

### 2.4. DNA Contamination

**Figure 3:**
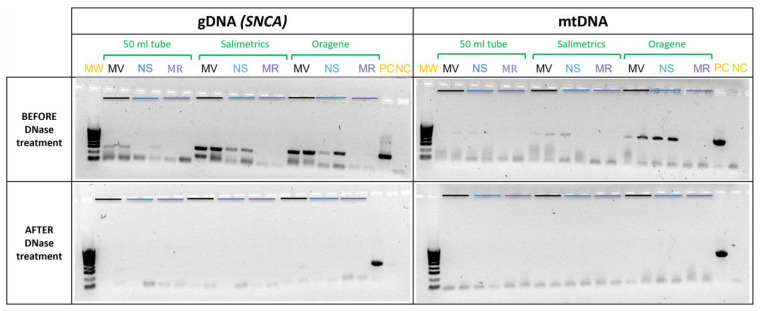
DNA contamination. 1.5% agarose gels showing the purity of the RNA obtained from the different combinations of saliva collectors (50 mL tube, Salimetrics and Oragene) with the miRNA extraction kits (MV, Ambion mirVana miRNA isolation kit; NS, Nucleospin miRNA Plasma kit; MR, miRNeasy Serum/Plasma Advanced kit) before and after DNase treatment. Amplification of an intronic SNP of SNCA gene (280 bp) and a non-coding mitochondrial (mtDNA) region (623 bp) indicates presence of DNA in the nucleic acid extraction. In each reaction, a positive (PC) and a negative control (NC) were included.

Although NS and MR kits include a DNase treatment step in the protocol, agarose gel electrophoresis performed after reverse transcription and amplification of the SNP rs2736990 and the non-coding mtDNA region showed the presence of DNA in miRNA and total RNA samples obtained by the MV and the NS kits, independent of the saliva collector used. In none of the RNA samples obtained with the MR kit, was DNA detected (Figure 3). An additional post-purification DNase treatment performed for samples obtained with the NS and MV kits removed all remaining DNA (Figure 3).

No significant differences were observed between miRNA expression levels in pre- and post-DNase-treated samples.

### 2.5. miRNA Quantification

#### 2.5.1. Synthetic Spike-In Standards

**Figure 4:**
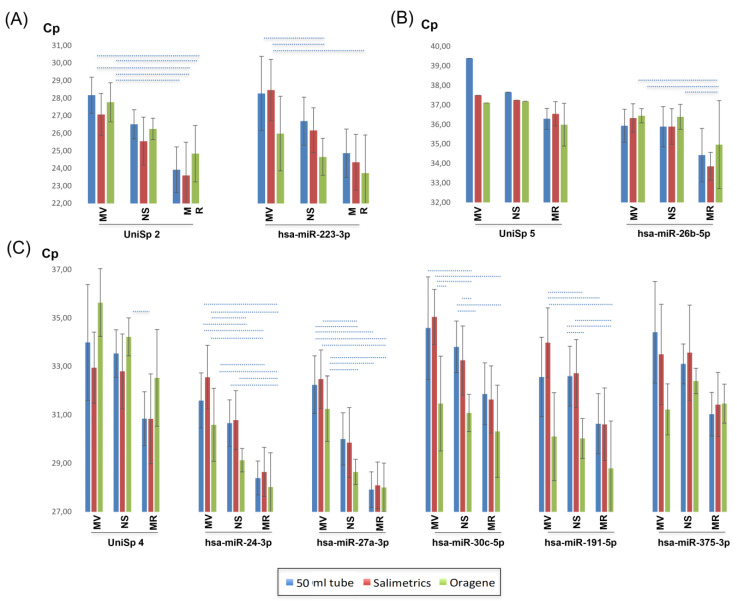
MiRNA expression levels are shown for all spike-in standards and hsa-miRNAs included in the study. (**A**): UniSp2 and hsa-miRNA with high expression, (**B**): UniSp5 and hsa-miRNA with low expression, (**C**): UniSp4 and hsa-miRNAs with intermediate expression. Dashed lines represent the most significant differences, *p* < 0.001.

The expression levels of UniSp3 and UniSp6 were constant, as observed with quantification cycle (Cq) values around 18, indicating reverse transcription reaction and qPCR worked correctly (Table S4). As expected, UniSp2, UniSp4 and UniSp5 presented different expression levels, on the one hand, due to their different concentrations and on the other, due to the different extraction efficiencies. UniSp2, as the most concentrated synthetic control, representing high expression, was detected between 23–28 cycles; UniSp4 between 30–36 cycles; and UniSp5, the most diluted and representing low expression, when detected it was between around 36–39 cycles (Figure 4).

When comparing the saliva collectors, in general, Salimetrics collectors yielded the highest expression corresponding to the lowest Cq values for concentrated spike-ins UniSp2 and UniSp4, being statistically significant for the latter (*p* < 0.001) (Figure 4A,C); however, the use of Oragene allowed to obtain constant expression data for the diluted spike-in UniSp5 (Figure 4B, Table 1). When comparing the extraction kits, significant differences were observed among them for the three spike-ins (*p* < 0.001 for UniSp2, *p* < 0.001 for UniSp4 and *p* < 0.008 for UniSp5). In all cases, significantly higher expression corresponding to lower Cq values was observed when using the MR kit (Figure 4, Table 1 and Table 2). The use of the Oragene collector seemed to partially affect the stability of synthetic spike-in standards, except that of low expression UniSp5. whereas the hsa-miRNAs purified from saliva collected with Oragene were detected at fewer cycles than hsa-miRNAs collected with either 50 mL tubes or Salimetrics, UniSp2 and especially UniSp4 were detected later. Although this tendency is clearly reflected on expression charts, differences were not significant.

#### 2.5.2. Gender

The expression data were first normalized against the expression of the synthetic UniSp3 and UniSp6 spike-in standards. No differences in miRNA expression levels were detected between males and females; thus, the five samples were considered as one group.

#### 2.5.3. Quantification of Saliva miRNAs

All combinations of saliva collectors and miRNA extraction kits classified the average expression levels of the different miRNAs consistently as high-, intermediate- or low-expressing miRNAs. Correspondingly, expression levels of hsa-miR-223-3p were similar to those obtained for UniSp2, classifying hsa-miR223-3p as high expression. Hsa-miR-24-3p, hsa-miR-27-3p, hsa-miR-30c-5p, hsa-miR-191-5p and hsa-miR-375-3p presented intermediate expression, with Cq values similar to UniSp4 Cq values. Low expression, with Cq values similar to UniSp5 Cq values, was found for hsa-miR-26b-5p (Figure 4).

Figure 4 contains a detailed representation of all expression results. The most pronounced expression differences (*p* < 0.001) are indicated, and all significant differences are summarized in Table S5.

These results seem to depict clear tendencies towards better performance of Oragene collectors on one hand, and of the MR extraction kit on the other. Therefore, miRNA expression was analyzed grouped by the collectors and by the extraction kits. First, ANOVA analysis showed significant differences between collectors in all analyzed miRNAs except for hsa-miR-26b-5p (*p* < 0.0001 for hsa-miR-30c-5p, *p* < 0.0001 for hsa-miR-191-5p, *p* < 0.0001 for hsa-miR223-3p and hsa-miR-24-3p, and *p* < 0.02 for hss-miR-27a-3p and hsa-miR-375-3p). No significant differences were found between 50 mL tubes and Salimetrics (Table 3); however, the use of the Oragene collector resulted in the detection of significantly higher expression levels of four out of the seven analyzed miRNAs, thereby being the collector that obtained better results. When collected with Oragene, for hsa-miR-223-3p, expression was detected 1.7 and 1.5 cycles earlier than with 50 mL tubes and Salimetrics, respectively; for hsa-miR-24-3p 1.4 cycles earlier than with Salimetrics; and for hsa-miR-191-5p and hsa-miR-30c-5p more than 2.2 cycles earlier than with both 50 mL tubes and Salimetrics. Although hsa-miR-375-3p hsa-miR-27a-5p and miR-26b-5p were also detected earlier when collected with Oragene, these differences were not significant.

On the other hand, ANOVA analysis also showed significant differences among extraction kits in all the analyzed miRNAs (*p* < 0.0001 for hsa-miR-24-3p, *p* < 0.0001 for hsa-miR-27a-3p, *p* = 0.0004 for hsa-miR-223-3p, *p* < 0.0001 for hsa-miR-30c-5p, *p* < 0.0001 for hsa-miR-191-5p and *p* = 0.0004 for hsa-miR375-3p), including for the low expression miRNA, hsa-miR-26b-5p (*p* < 0.0001). Two miRNAs were detected earlier when extracted with MV compared with NS, hsa-miR-24-3p, 1.4 cycles; and hsa-miR-27a-3p, 2.5 cycles. When comparing MV and NS with MR, only hsa-miR-375-3p did not show significant expression difference when extracted with MV compared to MR (Table 3).

When comparing all collector-miRNA extraction kit combinations, again, in all cases, significantly higher expression was observed when using the MR kit (Figure 4, Table S5).

## 3. Discussion

The main aim of this study was to identify the best combination of saliva collectors and miRNA extraction kits, allowing us to obtain an adequate quantity and quality of miRNA with an easily reproducible protocol. To achieve our goal, we evaluated several features. On one hand, we analyzed parameters related to RNA quality, such as amount and purity. On the other hand, technical parameters, including the time cost and complexity of the protocol, the economic cost of the different combinations and the power of recovering low-expression miRNAs were taken into account. To avoid any possible cross-contamination from potential external miRNA sources, all samples were collected after 30 min minimum of having eaten, drunk, smoked or chewed gum. All samples were collected in the same time range to avoid diurnal oscillations [8], and participants were selected from the same age range to minimize the effect of aging on miRNA expression [5].

**Figure 5:**
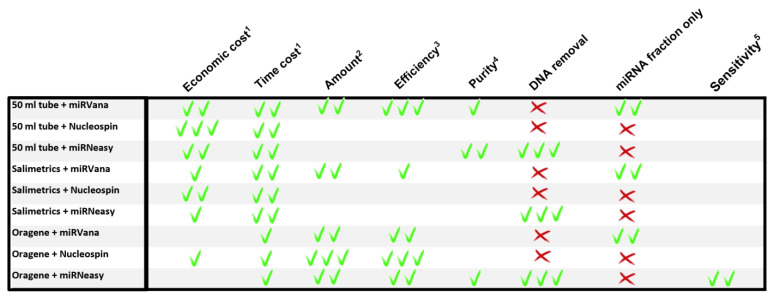
Schematic summary of the different features evaluated. A green tick means achieved; red cross means not achieved. The number of ticks represents the grade of achievement. When referring to the economic cost, one tick represents a more expensive combination than two or three ticks. One tick for time cost represents longer processing times. ^1^ per miRNA extraction, ^2^ total RNA amount (ng), ^3^ ng total RNA/ ul saliva, ^4^ A260/280, ^5^ potential of extracting low expressing miRNAs.

The result of analyzing the different features with respect to miRNA expression studies in saliva is summarized in Figure 5.

In the present study, DNase treatment was carried out in most cases after the RNA purification procedure because DNA was detected in RNA samples purified with both the MV and NS kits. Although the NS kit includes DNase, the recommended treatment did not efficiently remove the DNA, probably due to the intended use of the kit to extract cell-free RNA from plasma (NucleoSpin^®®^ miRNA Plasma). Although the additional DNase treatment seemed not to affect miRNA quantification, when extracting RNA from saliva, the treatment with DNase should be included in the main RNA extraction protocol. The elimination of residual DNA, which has an elevated inter-individual heterogeneity, not only avoids interference during concentration determination but will also assure RNA quality for eventual mRNA expression studies.

Synthetic spike-in standards of high and intermediate expression, UniSp2 and UniSp4, seemed to degrade when saliva was obtained with Oragene collectors. The Oragene manufacturer protocol indicates that after the two-hour incubation at 50 °C, and before the RNA purification, a neutralizer solution should be added to the saliva sample. We did not include this step in our purification protocol and cannot affirm that the lack of the neutralizer solution is responsible for the apparent degradation, especially because miRNA expression was not affected. On the contrary, in virtually all cases, miRNA expression was highest using the Oragene collector. Since synthetic spike-in standards are used to normalize miRNA purification quality, this capacity would not be affected at lower detection rates.

Here we refer to major or minor expression detection of the different miRNAs or spike-in controls. However, this is translated into the capability to extract the totality of miRNAs contained in the saliva sample. Oragene seemed to be the best collector to extract the major amount of RNA, and the most efficient, although the purity obtained was not the best. Nonetheless, it was the most expensive collector and required a longer RNA/miRNA purification time. Independently of the miRNA extraction kit, Oragene obtains low expression miRNAs at sufficient concentration to be analyzed within a reproducible expression range. One possible reason is the RNA-stabilizing solution included in the collector kit itself. The importance of RNA stabilizers has been reported recently in a study which addressed the identification of confounding factors in saliva-based miRNA studies [22]. Less variability was detected for those saliva samples that were collected in the presence of an RNA stabilizer. In our study, no significant differences in the inter-individual variability using the different collectors, kits or their combinations were found, probably due to the reduced sample number. On the other hand, the MR kit seemed to be the only kit allowing DNA-free RNA extraction. Additionally, although Oragene collectors did not extract the major amount of RNAs most efficiently, its combination with the MR kit obtains completely DNA-free low expression miRNAs with the best yields. This combination was able to obtain the highest expression of most of miRNAs analyzed, and extract UniSp5 in more than one sample, and most hsa-miR-26b-5p representing a low-expression miRNA.

However, the selection of the best collector and extraction kit depends on the aim of each study. For instance, if there is the need to obtain small RNAs only, the MV kit would be the adequate option. Of the three RNA purification kits, MV is the only one that permits the separation of small RNAs from total RNA during the purification procedure. Additionally, the MV kit in combination with 50 mL tubes for saliva collection seems to be an optimal choice for this purpose (Figure 5).

Our results also underline that planning each experiment is mandatory. When working in a context of no restricted financing, all experiments can be carried out in the same conditions using the combination of Oragene/MR. These samples will be of a quality that allows a large number of analyses to be carried out over a longer period of time. But if there is restricted financing, each experiment should be designed in view of the expected results. For example, if a validation study will include a defined sample number and is carried out within a defined time frame, the MR kit could be combined with 50 mL tubes, drastically reducing both time and economic cost of the study.

The miRNAs we included in our study were selected according to their previously reported expression levels [23]. However, several of these miRNAs were classified to express in a different range in our study, e.g., for hsa-miR-191-5p reported as high expression, and hsa-miR-30c-5p and hsa-miR-375-3p reported as low expression miRNAs. This difference could be due to the different protocols used, although we used several protocols combining different sampling and purification options and all miRNA were classified similarly as low-, intermediate- or high expression. Another cause could be specific characteristics of the population and/or individuals included here.

It is now widely accepted that assuring the quality of research and following minimal requirements in research practice is mandatory. For several years now, there has been an open discussion about repeated problems with the reproducibility of scientific results in almost all fields of research [24,25]. The concerns were addressed by specialists in several fields [26], and an extensive debate on the topic has resulted in established guidelines for Good Research Practice [27]. These guidelines contain simple rules that can be grouped into three main sections: planning, execution, and reporting [28]. Specifically, the MIQE guidelines show that the results obtained in studies based on quantitative real-time PCR experiments should be reported providing certain experimental information [29], and in the field of miRNA-related research, the strict application of these guidelines is also mandatory. Several studies addressing the optimization of total RNA purification from saliva samples have been carried out [13,14,30,31]. On one hand, studies reported by Ostheim and colleagues have specifically investigated the influence of the microbiome on saliva RNA expression [13,14]. In their studies, the results indicated that with higher microbiome content, there is an increasing inhibition of RNA expression. To overcome this problem, previous to cDNA synthesis, RNA concentration was adjusted, taking into account bacterial 16S rRNA [14]. In another study, the lysis protocol of a TRIzol-based RNA extraction method was modified to improve both RNA quality and yield [31]. To test the outcome of the improvement, four mRNAs but no miRNAs were quantified. In this study, RNA was extracted from saliva pellets, and no comparison with the cell-free RNA content was performed. Finally, another study addressed the effect of using different RNA stabilizers during saliva collection and concluded that the use of such stabilizers results in the obtaining of high-yield and high-quality RNAs [30]. Although in our study miRNA expression quality was higher for four out of the seven analyzed miRNAs, we found that the impact of choosing the RNA extraction kit was higher than of the collection tube. Cq values representing expression levels were higher for miRNAs extracted with the MR kit than with the MV or NS.

Altogether, the results of these studies indicate the MIQE guidelines should probably be extended to address specific circumstances arising from the different biological fluids and content (miRNA, lncRNA, mRNA) to be detected.

Our study has several limitations. Although cleared salivary supernatant is preferable [32] for RNA purification, here we isolated total and miRNA from whole saliva. Neither had we obtained an exosome-enriched fraction previous to RNA purification since the isolation of exosomes results in low RNA yields [13]. Another variable we did not address in this study is that the oral microbiome represents a significant source of the total and miRNAs obtained from whole saliva [33,34,35]. In NS as well as MR kits, the final nucleic acid obtained is total RNA, including small RNAs. As we did not know the exact proportion of miRNAs included in the final elution, the quantification was considered as total RNA (A260 × 40), assuming that in a final accrued assessment, the total value would be inferior (A260 × 40 + A260 × 33). Finally, we were able to include only a very few individuals in this study. Therefore, the effect of the different collector/extraction kit combinations on intra- or inter-individual variability could not be assessed.

## 4. Materials and Methods

### 4.1. Participants

Saliva samples were obtained from five volunteers, three females and two males, all of them 36–56 years old. Since it has been reported that miRNA expression suffers essential changes after three aging peaks produced around the ages of 34, 60 and 75 years [6], participants from the same aging group were recruited to minimize inter-individual heterogeneity.

The 15 saliva samples were collected in the same time range (8:00–10:00 AM) on three different days, to avoid fluctuations as described for circadian miRNAs in plasma and saliva [7,8]. Although lifestyle-related factors, such as smoking and exercise have also been described to alter plasma miRNA profiles [10,11], we were not able to adjust the samples for these factors.

### 4.2. Sample Collection

The saliva samples were collected by passive drool, using the three different receptacles: (1) Oragene RNA (DNA Genotek, Ottawa, ON, Canada), a kit to collect 2 mL of saliva containing RNA stabilizer solution. After collection, saliva samples were mixed with the stabilizer by capping the vial followed by vigorous shaking of the capped vial for 10 s, and stored at room temperature. (2) SalivaBio’s 2 mL cryovials and the Saliva Collection Aid (exclusively from Salimetrics, State College, PA, USA) are designed to improve volume collection: once the ribbed-end of the Saliva Collection Aid was placed securely into the collection tube, saliva was guided through that aid into the vial and samples were immediately stored at −80 °C. (3) 50 mL sterile conical tubes, where the participants just drool around 2 mL of saliva into the tube, were immediately stored at −80 °C. In order to avoid any possible cross-contamination from external sources of miRNA, all samples were collected after 30 min minimum of having eaten, drunk, smoked or chewed gum.

### 4.3. miRNA Extraction

Three different kits were used: (1) Ambion mirVana miRNA isolation kit (ThermoFisher Scientific, Waltham, MA, USA) (abbreviated as MV) which isolates total RNA and small RNAs (<200 nt) in two different elutions from 20 samples. (2) NucleoSpin^®®^ miRNA Plasma, version November 2018/Rev. 06 (Macherey-Nagel, Hoerdt, France) (abbreviated as NS) which isolates total RNA, including small RNA and DNA from plasma from 50 samples; it includes an optional DNA digestion, which was performed because saliva is a DNA-rich biofluid. (3) miRNeasy Serum/Plasma Advanced Kit, version January 2020 (Qiagen, Hilden, Germany) (abbreviated as MR) which purifies total RNA, including miRNA from 50 samples. In this kit, there is an optional on-column DNase digestion using the RNase-Free DNase Set, version June 2018 (Qiagen) (not included in the kit) that was also used in the miRNA extraction procedure.

For all three kits, manufacturer’s instructions were followed adding a previous incubation to the samples that had been collected with Oragene (50 °C in an air incubator during 2 h). The detailed protocols including specific modifications, such as the use of the maximum saliva volume and DNase digestion are provided in Appendix A. All kits are based on a first step of cell lysis and disruption. Whereas the MV kit includes an organic extraction, both the NS and MR kits follow phenol-free protocols. All three kits comprise several washings and a final RNA elution step. In the three cases, the maximum volume of saliva recommended by the manufacturer was used (500 µL for MV, 600 µL for MR and 900 µL for NS) increasing proportionally the volumes of the different reagents as recommended (see the Appendix A for detailed description). MiRNA-fractions and total RNA samples were eluted with 20 µL RNase-free water. Total RNA of the MV kit was eluted in 50 µL. In total, 60 RNA samples were obtained (Figure 1).

### 4.4. RNA Concentration and Purity

RNA concentration was quantified by a spectrophotometer (DeNovix Inc., Wilmington, DE, USA), reading the absorbance at 260 nm and 280 nm. RNA concentration was calculated in µg/mL as follows: total RNA ≈ 40 × A260, and miRNA ≈ 33 × A260, as suggested by the MV kit instructions. The total RNA amount was calculated by multiplying the concentration by the final sample volume. Purity was assessed by the A260/A280 ratio, considering high pure RNA those samples with a ratio between 1.8–2.1. RNA extraction efficiency was evaluated by the ratio of the amount of total RNA and the saliva volume initially processed according to each extraction protocol.

### 4.5. DNA Contamination Check

To verify that RNA samples were DNA-free, 250 ng of total RNA (or miRNA in case of the MV kit) were used for reverse transcription by Ready-to-goTM You-Prime First-Strand Beads (GE Healthcare, Uppsala, Sweden) and amplified by standard PCR that were carried out in 15 µL reactions with Biotaq DNA polymerase (Bioline, London, UK). An intronic SNP (rs2736990) located in the α-synuclein gene (SNCA) and a fragment of the non-coding mitochondrial DNA (mtDNA) region were amplified to test for DNA contamination. Primer sequences, fragment length and PCR conditions are shown in Table 4. A DNA sample extracted previously from peripheral blood was used as a positive and RNase-free water as a negative control. PCR products were electrophoresed on 1.5% agarose gels.

An additional DNase treatment was performed on those samples that contained DNA using the DNA-freeTM DNA Removal Kit (ThermoFisher) following the manufacturer’s instructions. After re-quantification of RNAs reverse transcription and amplification of rs2736990 and mtDNA were repeated.

### 4.6. Synthetic RNA Spike-In

In order to provide a control for the quality of the RNA isolation, miRNA cDNA synthesis and quantification, RNA spike-ins were added to the sample: (1) prior to RNA isolation: UniSp2, UniSp4 and UniSp5 were provided pre-mixed in one vial, each at a different concentration with 100-fold increments (miRCURY LNA RNA Spike-In Kit, Qiagen). One µL RNA spike-in mix was added to the lysis buffer before adding the sample (see detailed in Appendix A); (2) prior to cDNA synthesis: UniSp6 (miRCURY LNA RT Kit, Qiagen), 0.5 µL was added to the reverse transcription reaction; and (3) miRNA quantification: UniSp3 contained in the miRCURY^®®^ LNA^®®^ SYBR^®®^ Green PCR Kit (Qiagen) is used as inter-plate calibrator.

### 4.7. miRNA Quantification

Hsa-miRNAs and synthetic spike-in controls included in this study are listed in Table 5. In total, seven miRNAs previously reported to be expressed in saliva with possibly different levels [23] were selected to test the different miRNA extraction methods: (1) high expression miRNAs detectable between 19–24 cycles: hsa-miR-191-5p and hsa-miR-223-3p; (2) intermediate-expression miRNAs detectable between 24–30 cycles: hsa-miR-24-3p and hsa-miR-26b-5p, which have been also recommended as housekeeping miRNAs in saliva in a cancer-related study [36]; (3) low expression miRNAs detectable between 30–32 cycles: hsa-miR-30c-5p and hsa-miR-375-3p; and (4) hsa-miR-27a-3p as a reference for Parkinson’s disease [37].

Expression of miRNAs was quantified by miRCURY^®®^ LNA^®®^ miRNA Custom Panels (Qiagen) using a LightCycler 480 instrument (Roche Diagnostics, Mannheim, Germany). Ten ng of either total RNA or miRNA (obtained by mirVana, Thermo Fisher, Vilnius, Lithuania) were reversely transcribed following the manufacturer instructions of the miRCURY LNA Universal RT miRNA SYBR^®®^ Green PCR-kit (Qiagen). cDNAs were amplified as described by manufacturer using the miRCURY^®®^ LNA^®®^ SYBR^®®^ Green PCR- kit (Qiagen). DNA was used as a negative control to assure miRNA quantification only.

To account for inter- and intra-run variations, all experiments were performed in technical duplicates and UniSp3 was used as an interplate calibrator. Raw quantification cycles (Cq) were obtained by the LightCycler software. Cq of 40 or higher were considered as no-expression.

### 4.8. Data Analysis

Samples were analyzed in nine different groups according to the different combinations of collectors and extraction kits. To test differences in the amount and efficiency of extracted RNA between the different collectors or the different RNA extraction kits, one-way and two-way ANOVA tests were performed. Wilcoxon Signed Rank Test was applied to compare all combinations.

Cq data were normalized using spike-in 3 and 6. Since the normal distribution of the data cannot be guaranteed, the Wilcoxon Signed Rank test was performed for the analysis of normalized Cq in pre-treated samples compared with DNase-treated samples, and of miRNA and spike-in 2, 4 and 5 expression comparing groups of collectors, extraction kits and both. Additionally, when data from all samples obtained with the same collector or the same extraction kit were grouped, Cq-values were normalized using the group mean.

*p*-values less than 0.05 were considered as significant. However, when groups were defined considering both variables (collectors and extraction kits) together, the *p*-value was corrected for multiple comparison testing, and significance was established as significant at *p* < 0.025. All the statistical analyses were performed using R software (R Core Team 2021), version 3.6.1 for Windows.

## 5. Conclusions

The testing of different combinations of saliva collectors and RNA purification kits permitted identification of combinations for different uses. Whereas the MV kit obtains small RNAs in an independent fraction, low expressing miRNAs could be reliably detected using the combination of the Oragene collector and the MR kit. The quantification of highly expressing miRNAs within a well-defined study can be carried out in an inexpensive combination of 50 mL tube collectors and the NS kit. Thus, identifying the scope of each study and taking into account the expected outcome will make it easy to decide which methodology is best to be used.

## Figures and Tables

**Table 1:** Cq values for spike-in 2, 4 and 5 obtained using the different saliva collectors and miRNA extraction kit combinations.

Spike-In	miRNA Extraction Kit	Saliva Collector					
		50 mL Tube		Salimetrics		Oragene	
		Cq Average	Cq SD	Cq Average	Cq SD	Cq Average	Cq SD
UniSp2	MV	28.17	1.11	27.07	1.29	27.77	1.18
	NS	26.51	0.89	25.55	1.46	26.24	0.64
	MR	23.92	1.39	23.59	2.03	24.84	1.70
UniSp4	MV	33.99	2.58	32.95	1.57	35.63	1.54
	NS	33.54	1.05	32.79	1.65	34.22	0.83
	MR	30.85	1.19	30.84	1.97	32.53	2.10
UniSp5	MV	39.40	-	37.50	-	37.11	-
	NS	37.66	-	37.25	-	37.19	-
	MR	36.29	0.62	36.55	0.70	35.99	1.23

Values are shown as average values with standard deviation (SD).

**Table 2:** Comparison of the Cq values of Spike-in 2, 4 and 5 obtained by the different combinations of saliva collectors and miRNA extraction kits.

UniSp2										
		MV			NS			MR		
		50 mL ^1^	Salim. ^2^	Orag. ^3^	50 mL	Salim.	Orag.	50 mL	Salim.	Orag.
MV	50 mL		0.152	0.669	**0.0059**	**0.0022**	**0.0011**	**0.0014**	**0.0003**	**0.0001**
	Salim.			0.360	0.344	**0.0379**	0.068	**0.0038**	**0.0011**	**0.0062**
	Orag.				**0.0434**	**0.0044**	**0.0058**	**0.0006**	**0.0002**	**0.0001**
NS	50 mL					0.382	0.450	**0.0013**	**0.0047**	**0.0117**
	Salim.						0.351	**0.031**	0.083	0.460
	Orag.							**0.0025**	**0.0087**	0.064
MR	50 mL								0.713	0.3066
	Salim.									0.122
	Orag.									
**UniSp4**										
		**MV**			**NS**			**MR**		
		**50 mL**	**Salim.**	**Orag.**	**50 mL**	**Salim.**	**Orag.**	**50 mL**	**Salim.**	**Orag.**
MV	50 mL		0.418	0181	0.613	0.281	0.660	**0.020**	**0.205**	0.315
	Salim.			**0.0080**	0.382	0.795	0.068	**0.031**	0.083	0.897
	Orag.				**0.0080**	**0.0080**	0.049	**0.0024**	**0.0013**	**0.0018**
NS	50 mL					0.328	0.144	**0.0013**	**0.010**	0.460
	Salim.						**0.024**	**0.014**	**0.049**	0.897
	Orag.							**0.0004**	**0.0016**	**0.028**
MR	50 mL								0.958	0.068
	Salim.									0.083
	Orag.									
**UniSp5**										
		**MV**			**NS**			**MR**		
		**50 mL**	**Salim.**	**Orag.**	**50 mL**	**Salim.**	**Orag.**	**50 mL**	**Salim.**	**Orag.**
MV	50 mL		1	1	1	1	0.429	0.276	0.333	0.333
	Salim.			1	1	1	0.479	0.276	0.333	0.333
	Orag.				1	1	0.479	0.717	0.667	1
NS	50 mL					1	0.479	0.276	0.333	0.333
	Salim.						0.479	0.276	0.667	0.667
	Orag.							0.095	0.329	0.845
MR	50 mL								0.539	0.712
	Salim.									0.421
	Orag.									

The Wilcoxon Signed Rank test was used. In red, *p*-values between 0.025 and 0.05 (clear tendency to significance); in green, statistically significant *p*-values (*p* < 0.025). ^1^ 50 mL tube, ^2^ Salimetrics, ^3^ Oragene.

**Table 3:** Comparison of miRNA expression in grouping the results by collectors and extraction kits.

		miR-223-3p	miR-24-3p	miR-191-5p	miR-30c-5p	miR-375-3p	miR-27a-3p	miR-26b-5p
Coll. ^1^	50 mL ^3^ vs. Salim. ^4^	0.992	0.377	0.728	0.958	0.744	0.808	0.601
	50mL vs. Orag. ^5^	<0.0001	0.015	<0.0001	<0.0001	0.131	0.404	0.573
	Salim. vs. Orag.	0.0002	0.0097	<0.0001	<0.0001	0.893	0.608	0.866
Kit ^2^	MV vs. NS	0.116	0.0023	0.978	0.651	0.409	<0.0001	0.614
	MV vs. MR	<0.0001	<0.0001	0.0028	0.0026	0.440	<0.0001	<0.0001
	NS vs. MR	<0.0001	<0.0001	0.0003	0.0008	0.0014	0.0031	<0.0001

The Wilcoxon Signed Rank test was used. All *p*-values are shown. In green, statistically significant *p*-values (*p* < 0.05). ^1^ Collectors, ^2^ Purification Kit, ^3^ 50 mL tube, ^4^ Salimetrics, ^5^ Oragene.

**Table 4:** Assessment of DNA contamination. Primer sequences, amplicon length and PCR conditions.

Primer Name	Primer SEQUENCE (5′->3′)	Length (bp)	Denaturation Temp/Time	Annealing Temp/Time	Extension Temp/Time	Cycles
rs2736990 PrU	TGGCAGTTGAGAGGAGTATTC	280	95 °C/40″	62 °C/30″	72 °C/50″	35
rs2736990 PrL	GTGACTAGCAGATGATGAGCA					
L2-16485	GAACTGTATCCGACATCTGG	560	94 °C/60″	55 °C/40″	72 °C/60″	35
H2-481	GATTAGTAGTATGGGAGTGG					

**Table 5:** Hsa-miRNA sequences and synthetic spike-in controls analyzed in this study.

	Cat. Nº MiRCury Assay	miRNA ID	Target Sequence
1	YP00205986	hsa-miR-223-3p	UGUCAGUUUGUCAAAUACCCCA
2	YP00204260	hsa-miR-24-3p	UGGCUCAGUUCAGCAGGAACAG
3	YP00204306	hsa-miR-191-5p	CAACGGAAUCCCAAAAGCAGCUG
4	YP00204783	hsa-miR-30c-5p	UGUAAACAUCCUACACUCUCAGC
5	YP00204362	hsa-miR-375-3p	UUUGUUCGUUCGGCUCGCGUGA
6	YP00206038	hsa-miR-27a-3p	UUCACAGUGGCUAAGUUCCGC
7	YP00204117	hsa-miR-26b-5p	CCUGUUCUCCAUUACUUGGCUC
8	YP00203950	UniSp2	
9	YP00203953	UniSp4	
10	YP00203955	UniSp5	
11	YP02119288	UniSp3	
12	YP00203954	UniSp6	

## Data Availability

Additional data can be requested from the corresponding author of this study.

## Supplementary Materials

The following supporting information can be downloaded at: https://www.mdpi.com/article/10.3390/ijms24032386/s1.

## Appendix A

**miRNA isolation using the Ambion mirVana miRNA Isolation Kit**

1. Thaw the frozen samples (if saliva was collected by Oragene collector, incubate the entire sample in the original vial at 50 °C for 2 h in an air incubator).
2. Add 1 μL Spike-in to 600 μL Lysis Buffer and mix by pipetting.
3. Add the mix of Lysis Buffer with Spike-in to 500 μL of saliva sample.
4. Add 50 μL of miRNA Homogenate Additive (1/10 volume), mix well by inverting the tubes several times.
5. Leave the mixture on ice for 10 min. During this time, prepare two new 1.5 mL tubes.
6. Split the sample to the new tubes (around 550 μL in each tube) and add 500 μL (250 μL in each tube) of Acid-Phenol:Chloroform (a volume that is equal to the volume before addition of the Homogenate Additive).
7. Vortex for 1 min and centrifuge for 10 min at max speed.
8. Transfer the supernatant to new fresh tubes. At this point put in DEPC-treated water at 95 °C for use in eluting the RNA from the filter at the end of procedure.
9. Add 1/3 volume room temperature 100% EtOH to the aqueous phase, invert tubes several times to mix.
10. Place a filter cartridge into collection tube, pipet the mixture from the previous step onto the filter cartridge (up to 700 μL).
11. Centrifuge for approx. 15 s at min. 10,000× *g*, collect the filtrate (contains the miRNA).
12. Add 2/3 volume room temperature 100% EtOH to the filtrate, mix throughly.
13. Place a new cartridge into collection tube and pipette the filtrate/EtOH mixture onto a second filter cartridge.
14. Centrifuge for around 15 s at equal or less than 10,000× *g*, discard the flow-through, and reuse the collection tube for the washing steps.
15. Add 700 μL of Washing Solution 1 to the filter cartridge and centrifuge for around 5–10 s at equal or less than 10,000× *g*. Discard the through-flow, reuse the collection tube.
16. Add 500 μL of Washing Solution 2/3, centrifuge for 5–10 s at equal or less than 10,000× *g*.
17. Repeat with a second 500 μL of Washing Solution 2/3.
18. After discarding the flow-through, replace the filter in the same collection tube and spin for 1 min to remove residual liquid from the filter.
19. Transfer the filter into a new collection tube and apply 20 μL of pre-heated (60 °C) water to the center of the filter. Spin 1 min at max speed.
20. Store collected miRNA at −20 °C.

**miRNA isolation using the miRNeasy Serum/Plasma Advanced kit**

1. Thaw the frozen samples (if saliva was collected by Oragene collector, incubate the entire sample in the original vial at 50 °C for 2 h in an air incubator).
2. Add 180 μL Buffer RPL and 1 μL Spike-in into a 1.5-mL microcentrifuge tube
3. Transfer 600 μL saliva into the 1.5 mL microcentrifuge tube with the buffer and spike-in. Close the tube caps and vortex for > 5 s. Leave at room temperature for 3 min.
4. Add 60 μL Buffer RPP. Close the tube caps and mix vigorously by vortexing for >20 s. Incubate at room temperature for 3 min.
5. Centrifuge at 1200× *g* for 3 min at room temperature to pellet the precipitate. (Supernatant should be clear and colorless).
6. Transfer supernatant to a new microcentrifuge tube. Add 1 volume of isopropanol. Mix well by vortexing.
7. Transfer the entire sample to an RNeasy UCP MinElute column. Close the lid, and centrifuge for 15 s at ≥8000× *g*. Discard the flow-through.
8. Add 350 μL Buffer RWT (prepared with isopropanol) to the RNeasy UCP MinElute spin column. Close the lid gently, and centrifuge for 15 s at ≥8000× *g* to wash the membrane. Discard the flow-through. Reuse the collection tube in the next step.
9. Add 10 μL DNase I stock solution to 70 μL Buffer RDD. Mix by gently inverting the tube, and centrifuge briefly to collect residual liquid form the sides of the tube.
10. Add 80 μL of the DNase I incubation mix directly to the RNeasy UCP MinElute spin column membrane, and place on the benchtop (20–30 °C) for 15 min.
11. Add 500 μL Buffer RWT (prepared with isopropanol) to the RNeasy UCP MinElute spin column. Close the lid gently, and centrifuge for 15 s at ≥8000× *g*. Save the flow-through for use in step the next step.
12. Place the spin column in a new 2-mL collection tube. Apply the flow-through saved in the previous step to the spin column. Centrifuge for 15 s at ≥8000× *g*. Discard the flow-through. Reuse the collection tube in the next step.
13. Pipet 500 μL Buffer RPE onto the RNeasy UCP MinElute spin column. Close the lid, and centrifuge for 15 s at ≥8000× *g*. Discard the flow-through. Reuse the collection tube in the next step.
14. Add 500 μL of 80% ethanol to the RNeasy UCP MinElute spin column. Close the lid gently, and centrifuge for 2 min at ≥8000× *g* to wash the spin column membrane. Discard the flow-through. After centrifuge, remove the RNeasy UCP MinElute spin column from the collection tube so that the column does not contact the flow-through.
15. Place the RNeasy UCP MinElute spin column in a new 2-mL collection tube. Open the lid of the spin column and centrifuge at full speed for 5 min to dry the membrane. Discard the flow-through and the collection tube.
16. Place the RNeasy UCP MinElute spin column in a new 1.5 mL collection tube. Add 20 μL of pre-heated (60 °C) RNase-free water directly to the center of the spin column membrane and incubate 1min. Close the lid, and centrifuge for 1 min at full speed to elute the RNA.
17. Store collected miRNA at −20 °C.

**miRNA isolation using NucleoSpin^®®^ miRNA Plasma**

1. Thaw the frozen samples (if saliva was collected by Oragene collector, incubate the entire sample in the original vial at 50 °C for 2 h in an air incubator).
2. Add 270 μL Buffer MLP and 1 μL Spike-in into a 1.5 mL microcentrifuge tube.
3. Add 900 μL saliva sample and vortex for 5 s. Incubate for 3 min at room temperature.
4. Add 90 μL Buffer MPP and vortex for 5 s. Incubate for 1 min at room temperature. Centrifuge for 3 min at 11,000× *g* to pellet the protein.
5. Transfer the clear supernatant to two new collection tubes (approx. 600 μL per each tube).
6. Add 1200 μL isopropanol (600 μL per each tube) and vortex for 5 s.
7. Place a NucleoSpin^®®^ miRNA Column into a collection tube and load the sample onto the column.
8. Incubate for 2 min at room temperature and centrifuge for 30 s at 11,000× *g*. Discard the flow-through and place the column back into the collection tube. Repeat this step until all sample is loaded onto the column.
9. Add 700 μL Buffer MW2 to the NucleSpin^®®^ miRNA Column. Centrifuge for 30 s at 11,000× *g*. Discard flow-through and place the column back into the collection tube.
10. Add 250 μL Buffer MW2 to the NucleSpin^®®^ miRNA Column. Centrifuge for 2 min at 11,000× *g*. It is not necessary to discard the flow-through.
11. Add 50 μL rDNase (previously dissolved in Reaction Buffer) directly onto the silica membrane of the NucleSpin^®®^ miRNA Column. Close the lid and incubate at room temperature for 15 min.
12. Add 100 μL Buffer MW1 to the NucleSpin^®®^ miRNA Column. Centrifuge for 30 s at 11,000× *g*. Discard flow-through and place the column back into the collection tube.
13. Add 700 μL Buffer MW2 to the NucleSpin^®®^ miRNA Column. Centrifuge for 30 s at 11,000× *g*. Discard flow-through and place the column back into the collection tube.
14. Add 250 μL Buffer MW2 to the NucleSpin^®®^ miRNA Column. Centrifuge for 2 min at 11,000× *g* to dry the membrane completely.
15. Place the NucleSpin^®®^ miRNA Column in a new collection tube. Add 20 μL of pre-heated (60 °C) RNase-free H2O directly onto the silica membrane of the column. Incubate for 1 min at room temperature. Close the lid and centrifuge for 1 min at 11,000× *g*.
16. Store collected miRNA at −20 °C.
