# Supplementary material for: Obtaining miRNA from Saliva—Comparison of Sampling and Purification Methods
Source: Int J Mol Sci. 2023 Jan 25;24(3):2386. doi: 10.3390/ijms24032386 (PMC9916721; doi:10.3390/ijms24032386)
Supplement: Supplementary file 1 [file ijms-24-02386-s001.zip › ijms-2100802-supplementary.pdf]

Supplementary material

Table S1. Values obtained for each individual RNA purification, including amount, purity and efficiency.

Collector	Kit	Total Volum (µl)	Total RNA (ng)		$A_{260/280}$	Efficiency (ng total RNA/µl saliva)	collector	Kit	Total RNA (ng)		$A_{260/280}$	Efficiency (ng total RNA/µl saliva)	collector	Kit	Total RNA (ng)		$A_{260/280}$	Efficiency (ng total RNA/µl saliva)
50ml tube	MV	20	87.12	4207.1		8.41	Salimetrics	MV	29.04	457.04	1	0.91	Oragene	MV	514.8	1628.8	1.84	3.26
50ml tube	MV	50	4120		1.9		Salimetrics	MV	428		2.54		Oragene	MV	1114		2.22	
50ml tube	MV	20	10.56	798.56	0.32	1.60	Salimetrics	MV	510.84	1528.84	1.74	3.06	Oragene	MV	545.82	1355.82	1.55	2.71
50ml tube	MV	50	788		1.81		Salimetrics	MV	1018		1.36		Oragene	MV	810		2.15	
50ml tube	MV	20	275.88	4725.9	1.81	9.45	Salimetrics	MV	907.5	2017.5	1.8	4.04	Oragene	MV	2158.86	5108.86	1.86	10.22
50ml tube	MV	50	4450		1.8		Salimetrics	MV	1110		1.42		Oragene	MV	2950		2.04	
50ml tube	MV	20	506.22	1966.2	2.37	3.93	Salimetrics	MV	113.52	473.52	1.81	0.95	Oragene	MV	1716.66	2338.66	2.17	4.68
50ml tube	MV	50	1460		2.1		Salimetrics	MV	360		0.84		Oragene	MV	622		2.14	
50ml tube	MV	20	458.7	2870.7	2.03	5.74	Salimetrics	MV	1178.1	2078.1	1.85	4.16	Oragene	MV	939.84	1393.84	1.67	2.79
50ml tube	MV	50	2412		1.73		Salimetrics	MV	900		1.49		Oragene	MV	454		1.54	
			**Average**	**2913.7**		**5.83**			**Average**	**1311**		**2.62**			**Average**	**2365.2**		**4.73**
50ml tube	NS	20		1707.2	1.73	1.90	Salimetrics	NS		114.4	1.14	0.13	Oragene	NS		2786.4	1.98	3.10
50ml tube	NS	20		338.4	1.67	0.38	Salimetrics	NS		396.8	1.78	0.44	Oragene	NS		912	1.71	1.01
50ml tube	NS	20		1107.2	1.8	1.23	Salimetrics	NS		659.2	1.63	0.73	Oragene	NS		12029.6	1.62	13.37
50ml tube	NS	20		688	1.71	0.76	Salimetrics	NS		81.6	0.78	0.09	Oragene	NS		4294	1.85	4.77
50ml tube	NS	20		282.4	2.04	0.31	Salimetrics	NS		449.6	1.9	0.50	Oragene	NS		4991.2	1.58	5.55
			**Average**	**824.64**		**0.92**			**Average**	**340.32**		**0.38**			**Average**	**5002.64**		**5.56**
50ml tube	MR	20		512.8	1.83	0.85	Salimetrics	MR		0	2.25	0.00	Oragene	MR		542	2.08	0.90
50ml tube	MR	20		51.2	2.78	0.09	Salimetrics	MR		444.8	2.03	0.74	Oragene	MR		1624	1.78	2.71
50ml tube	MR	20		929.6	1.84	1.55	Salimetrics	MR		760.8	1.99	1.27	Oragene	MR		5144	1.9	8.57
50ml tube	MR	20		436.8	1.98	0.73	Salimetrics	MR		101.6	1.53	0.17	Oragene	MR		4469.6	1.72	7.45
50ml tube	MR	20		944.8	1.98	1.57	Salimetrics	MR		701.6	0.36	1.17	Oragene	MR		1398	0.83	2.33
			**Average**	**575.04**		**0.96**			**Average**	**401.76**		**0.67**			**Average**	**2635.52**		**4.39**

Table S2. P-values corresponding to the comparison of the total RNA amount obtained by the different combinations of saliva collectors and miRNA extraction kits applying the Wilcoxon Signed Rang test. In red, p< 0.05 (statistically not significant); in green, statistically significant p-values.

		50ml tube			Salimetrics			Oragene		
		MV	NS	MR	MV	NS	MR	MV	NS	MR
50ml tube	MV	-	**0,0317**	**0,0317**	0,151	**0,0079**	**0,0079**	0,6915	0,421	0,548
	NS	-	-	0,691	0,309	0,222	0,421	0,0556	**0,0317**	0,151
	MR	-	-	-	0,222	0,421	0,548	**0,0079**	**0,0317**	**0,0317**
Salimetrics	MV	-	-	-	-	**0,0317**	0,0952	0,421	0,556	0,421
	NS	-	-	-	-	-	0,841	**0,0079**	**0,0079**	**0,0159**
	MR	-	-	-	-	-	-	**0,0079**	**0,0079**	**0,0317**
Oragene	MV	-	-	-	-	-	-	-	0,421	1
	NS	-	-	-	-	-	-	-	-	0,309
	MR	-	-	-	-	-	-	-	-	-

The Wilcoxon Signed Rang test was used. In red, p< 0.05 (statistically not significant); in green, statistically significant p-values.

Table S3. P-values corresponding to the comparison of the miRNA extraction efficiency obtained by the different combinations of saliva collectors and miRNA extraction kits applying the Wilcoxon Signed Rang test. In red, p< 0.05 (statistically not significant); in green, statistically significant p-values.

		50ml tube			Salimetrics			Oragene		
		MV	NS	MR	MV	NS	MR	MV	NS	MR
50ml tube	MV	-	**0,0159**	**0,0079**	0,151	**0,00794**	**0,00794**	0,6905	0,691	0,548
	NS	-	-	1	0,0952	0,2222	0,5476	**0,00794**	**0,0317**	**0,0317**
	MR	-	-	-	0,0952	0,1412	0,5476	**0,00794**	**0,0317**	**0,0317**
Salimetrics	MV	-	-	-	-	**0,00794**	0,09524	0,4206	0,151	0,841
	NS	-	-	-	-	-	0,4206	**0,00794**	**0,00794**	**0,00794**
	MR	-	-	-	-	-	-	**0,00794**	**0,0317**	**0,03175**
Oragene	MV	-	-	-	-	-	-	-	0,691	0,463
	NS	-	-	-	-	-	-	-	-	0,691
	MR	-	-	-	-	-	-	-	-	-

The Wilcoxon Signed Rang test was used. In red, p< 0.05 (statistically not significant); in green, statistically significant p-values.

Table S4. Spike-in 3 and spike-in 6 Cp values obtained by the different saliva collectors and miRNA extraction kit combinations. Values are shown in Average and standard deviation (SD).

Spike-in	miRNA extaction kit	Saliva collector							
		50ml tube			Salimetrics			Oragene	
		Cq Average	Cq SD		Cq Average	Cq SD		Cq Average	Cq SD
UniSp3	MV	18,74	0,20		18,61	0,40		18,55	0,30
	NS	18,74	0,17		18,71	0,14		18,49	0,27
	MR	18,74	0,30		18,72	0,05		18,45	0,20
UniSp6	MV	18,56	0,56		18,39	0,55		18,29	0,19
	NS	18,37	0,29		18,26	0,36		18,38	0,39
	MR	18,24	0,22		17,97	0,14		18,57	0,56

Table S5. P-values corresponding to the comparison of the Cp values obtained from miRNA expression for the nine different combinations of collectors and extraction kits applying the Wilcoxon Signed Rang test. In red, p< 0.05 (statistically not significant); in green, statistically significant p-values.

**hsa-iR-223-3p**										
		MV			**NS**			**MR**		
		50ml[1]	Salim.[2]	Orag.[3]	50ml	Salim.	Orag.	50ml	Salim.	Orag.
MV	**50ml**		0,955	0,0635	0,336	0,0939	**0,0007**	**0,0059**	**0,0059**	**0,0046**
	**Salim.**			**0,0343**	**0,0499**	**0,0148**	**0,0006**	**0,0019**	**0,0011**	**0,00054**
	**Orag.**				0,3154	0,762	0,166	0,286	0,237	0,123
**NS**	**50ml**					0,574	**0,0086**	**0,0281**	**0,0148**	**0,00312**
	**Salim.**						**0,0343**	**0,0499**	**0,0281**	**0,0266**
	**Orag.**							0,829	0,356	0,529
**MR**	**50ml**								0,141	0,274
	**Salim.**									0,460
	**Oragene**									

**hsa-miR-24-3p**										
		MV			**NS**			**MR**		
		50ml	Salim.	Orag.	50ml	Salim.	Orag.	50ml	Salim.	Orag.
MV	**50ml**		0,189	0,161	0,152	0,336	**0,0001**	**0,00031**	**0,0031**	**0,0001**
	**Salim.**			**0,0266**	**0,0104**	**0,01041**	**0,00005**	**0,00031**	**0,001933**	**0,00005**
	**Orag.**				0,633	0,897	**0,0115**	**0,00055**	**0,008729**	**0,00072**
**NS**	**50ml**					0,721	**0,0021**	**0,00109**	**0,007362**	**0,00005**
	**Salim.**						**0,0014**	**0,00109**	**0,01003**	**0,00005**
	**Orag.**							**0,02052**	0,1418	**0,041**
**MR**	**50ml**								1	0,515
	**Salim.**									0,894
	**Oragene**									

**hsa-miR-191-5p**										
		MV			**NS**			**MR**		
		50ml	Salim.	Orag.	50ml	Salim.	Orag.	50ml	Salim.	Orag.
MV	**50ml**		0,1206	**0,0097**	0,867	0,955	**0,0097**	**0,0401**	**0,0205**	**0,0019**
	**Salim.**			**0,0025**	0,083	0,130	**0,0007**	**0,00062**	**0,0029**	**0,00005**
	**Orag.**				**0,0062**	**0,0062**	0,970	0,477	0,762	0,280
**NS**	**50ml**					0,798	**0,0009**	**0,0104**	**0,0207**	**0,00018**
	**Salim.**						**0,0016**	**0,0148**	**0,0148**	**0,00018**
	**Orag.**							0,307	0,450	0,140
**MR**	**50ml**								0,752	0,083
	**Salim.**									0,173
	**Oragene**									

**hsa-miR-30c-5p**										
		MV			**NS**			**MR**		
		50ml	Salim.	Orag.	50ml	Salim.	Orag.	50ml	Salim.	Orag.
MV	**50ml**		0,613	**0,0068**	0,613	0,354	**0,0001**	**0,0140**	**0,0093**	**0,0021**
	**Salim.**			**0,0006**	0,083	**0,021**	**0,00005**	**0,0011**	**0,0011**	**0,0006**
	**Orag.**				**0,01554**	**0,02052**	0,6842	0,5938	0,7221	0,273
**NS**	**50ml**					0,4418	**0,00009**	**0,01041**	**0,01476**	**0,0008**

	**Salim.**						**0,00055**	0,065	**0,0281**	**0,0029**
	**Orag.**							0,360	0,829	0,307
**MR**	**50ml**								0,328	0,068
	**Salim.**									0,351
	**Orag.**									

hsa-iR-375-3p

		MV			NS			MR		
		50ml	Salim.	Orag.	50ml	Salim.	Orag.	50ml	Salim.	Orag.
**MV**	**50ml**		0,628	**0,0075**	0,181	0,573	0,118	**0,0126**	**0,0293**	**0,0196**
	**Salim.**			0,0645	0,536	0,955	0,417	0,0721	0,0939	0,0874
	**Orag.**				**0,0014**	**0,0044**	**0,0147**	0,965	0,965	0,241
**NS**	**50ml**					0,959	0,101	**0,0011**	**0,0355**	**0,0018**
	**Salim.**						0,696	**0,010**	0,105	0,083
	**Orag.**							**0,0014**	0,237	**0,0091**
**MR**	**50ml**								0,674	0,286
	**Salim.**									0,625
	**Oragene**									

hsa-miR-27a-3p

		MV			NS			MR		
		50ml	Salim.	Orag.	50ml	Salim.	Orag.	50ml	Salim.	Orag.
**MV**	**50ml**		1	0,161	**0,0093**	**0,014**	**0,0008**	**0,00031**	**0,014**	**0,0001**
	**Salim.**			**0,034**	**0,0011**	**0,0029**	**0,0004**	**0,00015**	**0,0104**	**0,00005**
	**Orag.**				0,101	0,146	**0,0002**	**0,00005**	**0,0085**	**0,00001**
**NS**	**50ml**					0,959	**0,011**	**0,0011**	0,083	**0,0021**
	**Salim.**						0,154	**0,0019**	0,105	**0,016**
	**Orag.**							**0,0019**	0,398	0,212
**MR**	**50ml**								0,674	0,674
	**Salim.**									0,790
	**Oragene**									

hsa-iR-26b-5p

		MV			NS			MR		
		50ml	Salim.	Orag.	50ml	Salim.	Orag.	50ml	Salim.	Orag.
**MV**	**50ml**		0,429	0,333	0,667	0,889	0,267	0,178	**0,044**	0,909
	**Salim.**			0,945	0,534	0,628	0,950	**0,020**	**0,00067**	0,328
	**Orag.**				0,259	0,383	1	**0,014**	**0,00031**	0,252
**NS**	**50ml**					1	0,397	0,054	**0,0037**	0,681
	**Salim.**						0,281	**0,040**	**0,0037**	0,681
	**Orag.**							**0,0104**	**0,00015**	0,199
**MR**	**50ml**								0,574	0,236
	**Salim.**									0,059
	**Orag.**									

The Wilcoxon Signed Rang test was used. In red, p< 0.05 (statistically not significant); in green, statistically significant p-values. [1] 50 ml tube, [2] Salimetrics, [3] Oragene.
